# Intratympanic membrane cholesteatoma after traumatic tympanic membrane perforation: a case report

**DOI:** 10.1186/s13256-023-03757-9

**Published:** 2023-02-21

**Authors:** Junhui Jeong, Hyun Seung Choi

**Affiliations:** Department of Otorhinolaryngology, National Health Insurance Service Ilsan Hospital, 100 Ilsan-ro, Ilsandong-gu, Goyang, 10444 Korea

**Keywords:** Cholesteatoma, Intratympanic membrane cholesteatoma, Tympanic membrane, Tympanic membrane perforation

## Abstract

**Background:**

Intratympanic membrane cholesteatoma presents as an asymptomatic, white, round mass on the tympanic membrane, and is usually detected incidentally in children.

**Case presentation:**

A 12-year-old Korean boy visited our otorhinolaryngology clinic for a whitish mass on the right tympanic membrane. He had a history of traumatic tympanic membrane perforation in the right ear that had occurred 1 year prior, which had healed well with a paper patch placement. The mass was completely removed under local anesthesia during surgery with a microscope. The mass was on the outer epithelial layer of the right tympanic membrane and did not invade the middle fibrous and inner mucosal layers. Cholesteatoma was diagnosed on the basis of histopathology.

**Conclusion:**

Intratympanic membrane cholesteatoma may not induce symptoms or invade the middle ear because it can grow outwards into the external auditory canal. However, intratympanic membrane cholesteatoma can grow over time, and then after growth, it can compress the tympanic membrane and advance into the middle ear, which can cause symptoms such as hearing loss. Intratympanic membrane cholesteatoma in children should be carefully evaluated and followed, and surgical removal should be considered, even for asymptomatic cases, to minimize potential damage and hearing loss.

## Introduction

Congenital cholesteatoma in the middle ear is commonly observed in children [1]. However, cholesteatoma in the tympanic membrane is rare [2]. The condition presents as an asymptomatic, white, round mass on the tympanic membrane, and is usually detected incidentally [3]. Intratympanic membrane cholesteatoma (ITMC) can be divided into congenital or acquired categories. Acquired ITMC can occur due to inflammatory injury or surgery [4]. We present a case of a 12-year-old boy with ITMC after traumatic tympanic membrane perforation in the right ear.

## Case presentation

**Fig. 1:**
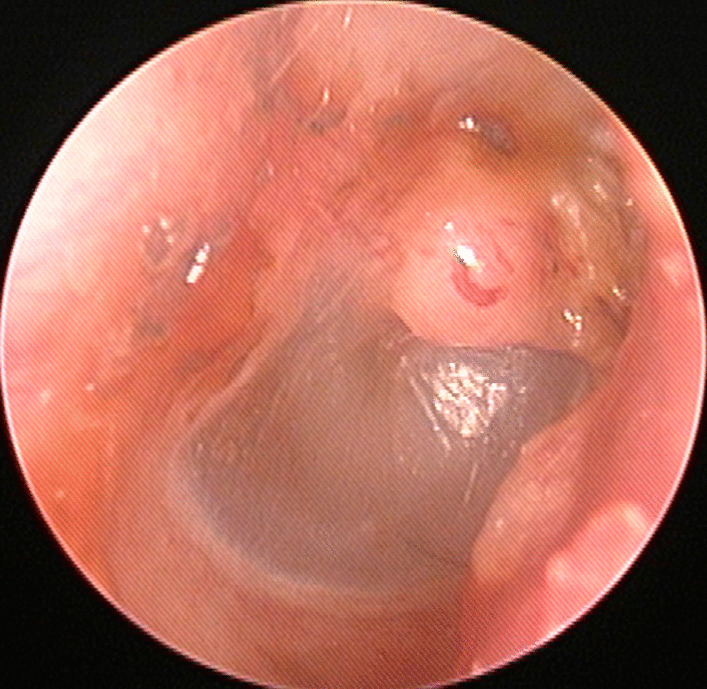
A whitish and spherical mass on the right tympanic membrane

**Fig. 2:**
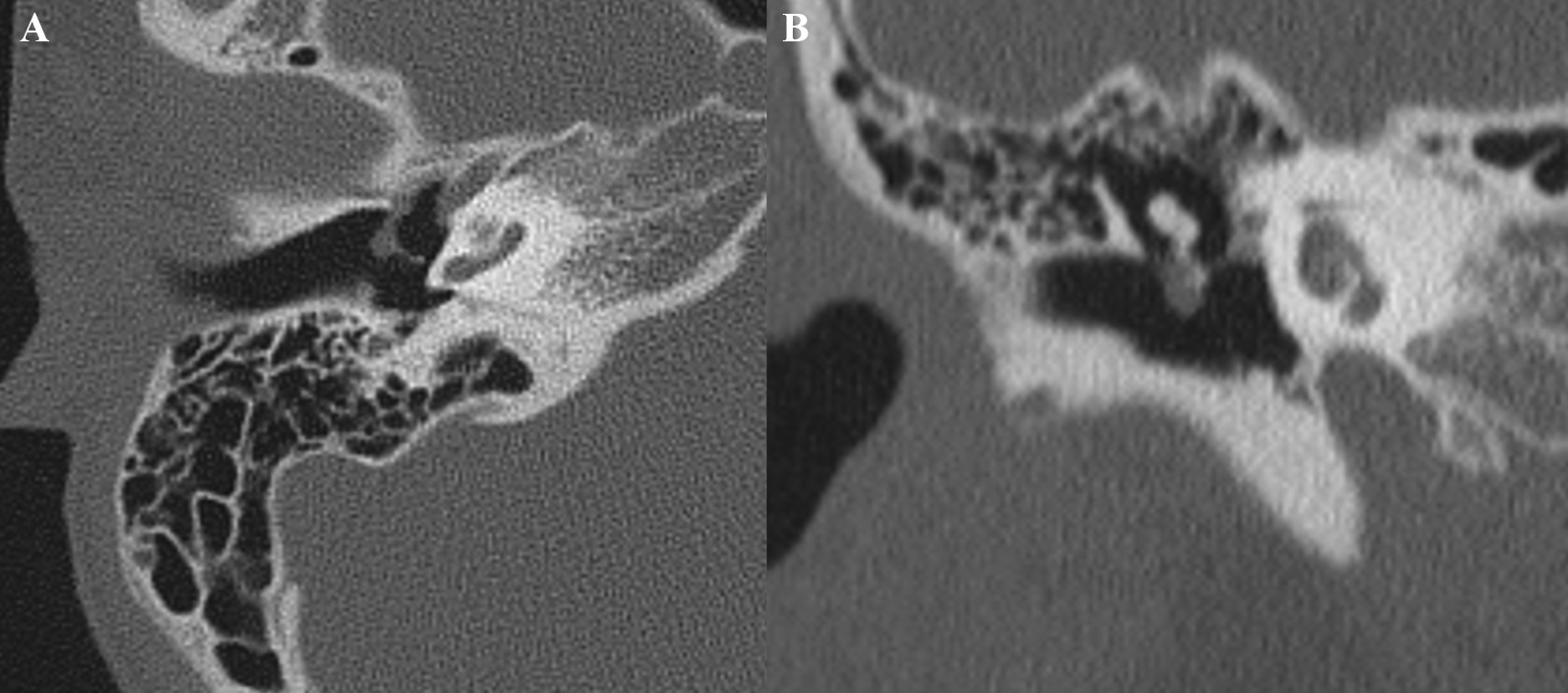
Temporal bone computed tomography revealing a 0.3 × 0.3-cm soft tissue density, cystic lesion in the right tympanic membrane, abutting on the malleus (**A** axial view, **B** coronal view)

A 12-year-old Korean boy visited our otorhinolaryngology clinic for a whitish mass on the right tympanic membrane, which was identified 1 month prior to presentation. He had neither a history of ear disease including recurrent otitis media or ear surgery including tympanostomy tube insertion in the right tympanic membrane nor any symptoms, including hearing loss. However, he had a history of traumatic tympanic membrane perforation in the right ear that had occurred 1 year prior, which had healed well with a paper patch placement performed within 1 week from the trauma. On physical examination, a whitish and spherical mass was observed on the right tympanic membrane (Fig. 1). Temporal bone computed tomography revealed a 0.3 × 0.3-cm soft tissue density which was a cystic lesion in the right tympanic membrane, abutting on the malleus (Fig. 2). Surgical excision was planned, considering the possibility of an intratympanic membrane cholesteatoma.

The mass was completely removed under local anesthesia during surgery with a microscope. The mass was on the outer epithelial layer of the right tympanic membrane and did not invade the middle fibrous and inner mucosal layers. Cholesteatoma was diagnosed on the basis of histopathology. The tympanic membrane healed well, and there was no recurrence or tympanic membrane perforation after 6 months.

## Discussion

ITMC was first reported by Hinton in 1863 [2, 5–7]. Since then, only a few cases have been reported, and the incidence is rare [2, 3]. ITMC is usually asymptomatic and is found incidentally in children. It typically appears as a round, spherical, white mass on the tympanic membrane [3].

The etiology of ITMC remains unclear [2, 6, 7]. ITMC can occur due to iatrogenic or traumatic tympanic membrane perforation. In patients with recurrent otitis media and local inflammation, metaplasia within the tympanic membrane may be induced, resulting in ITMC [2]. ITMC may develop from basal cell layer proliferation of the tympanic membrane into protruding cones due to an inflammatory process [4, 6].

However, in a systematic review by Ching *et al*., 51% of patients with ITMC had no history of previous otitis media. They reported that ITMC could be congenital because most patients were young children, with a mean age of 3 years, and there was a significant positive correlation between patient age and ITMC size, indicating that the congenital cholesteatoma gradually grew over time [1, 2]. Reports have suggested that the persistence of embryonic epithelial rests, which do not disappear after they contribute to tympanic membrane and tympanic ring development, could result in ITMC [6]. In this case, the patient had been aware of the ITMC after the traumatic tympanic membrane perforation and was 12 years of age at diagnosis. Therefore, this condition of ITMC may have developed after tympanic membrane perforation, rather than with congenital etiology. An association between ITMC and paper patch placement has not been reported. The paper patch placement in the present case seemed to have no effect on the formation of ITMC.

ITMC originates from the outer epithelial layer of the tympanic membrane, which causes the ITMC to grow outwards into the external auditory canal (EAC). Thus, invasion into the middle ear can be avoided [2]. Many reported cases of ITMC showed intact fibrous layer of the tympanic membrane and no middle ear invasion after enucleation of ITMC [1, 2, 5–7]. On the basis of these reports, surgical removal of ITMC may be delayed until 1 or 2 years of age [2]. ITMC may also spontaneously disappear if it ruptures into the EAC in the early stage and the keratin debris is completely discharged [7–9]. In comparison, several reports mentioned that surgical removal should be performed as soon as possible because ITMC could increase in size and expand into the middle ear [2, 6]. If ITMC would rupture or enlarge into the middle ear, it could result in middle ear cholesteatoma [8].

Surgical removal via a transcanal approach is recommended if the ITMC can be peeled off the tympanic membrane with an intact fibrous layer [2, 5, 7]. On the basis of a systematic review, most patients (93%) were treated with a transcanal approach [2]. Other studies reported using surgical removal with an endoscope [1], and carbon dioxide laser enabled ablation and resection without any endaural incision [10]. Recurrence of congenital ITMC is reported less frequently than middle ear cholesteatoma recurrence [5].

In practice, ITMC should be differentiated from diseases that present with similar findings, such as tympanosclerosis. Tympanosclerosis is characterized by calcified plaques that occur after myringotomy or tympanostomy tube insertion. Calcified plaques in the lamina propria appear as thin plates, whereas ITMC is spherical in shape [3]. After surgical excision, histopathologic examination is necessary to confirm the diagnosis.

## Conclusion

ITMC can occur in children, but is rare. ITMC may not induce symptoms or invade the middle ear because it can grow outwards into the EAC. However, ITMC can grow over time, and then after growth, it can compress the tympanic membrane and advance into the middle ear, which can cause symptoms such as hearing loss. Thus, ITMC in children should be carefully evaluated and followed, and surgical removal should be considered, even for asymptomatic cases, to minimize potential damage and hearing loss.

## Data Availability

Data sharing is not applicable to this article because no datasets were generated or analyzed during the current study.

## Abbreviations

ITMC: Intratympanic membrane cholesteatoma
EAC: External auditory canal
